# Natural killer cell-derived exosomes for cancer immunotherapy: innovative therapeutics art

**DOI:** 10.1186/s12935-023-02996-6

**Published:** 2023-08-05

**Authors:** Zahra Hatami, Zahra Sadat Hashemi, Mohamad Eftekhary, Ala Amiri, Vahid Karpisheh, Kaveh Nasrollahi, Reza Jafari

**Affiliations:** 1 Department of Immunology, Faculty of Medical Sciences, Tarbiat Modares University, Tehran, Iran; 2 ATMP Department, Breast Cancer Research Center, Motamed Cancer Institute, ACECR, Tehran, Iran; 3 Department of Medical Biotechnology, Faculty of Paramedicine, Guilan University of Medical Sciences, Rasht, Iran; 4 Department of Biotechnology, Faculty of Biological Sciences, Alzahra University, Tehran, Iran; 5 Immunology Research Center, Tabriz University of Medical Sciences, Tabriz, Iran; 6 Department of Biochemistry, Faculty of Biological Sciences, Tarbiat Modares University, Tehran, Iran; 7 Cellular and Molecular Research Center, Cellular and Molecular Medicine Institute, Urmia University of Medical Sciences, Urmia, Iran

**Keywords:** Natural killer cell, Exosomes, NK-derived Exosomes, CAR-NK, Cancer immunotherapy, Fas/FasL pathway, Immunostimulation

## Abstract

Chimeric antigen receptor natural killer cells (CAR-NK) promote off-the-shelf cellular therapy for solid tumors and malignancy.However,, the development of CAR-NK is due to their immune surveillance uncertainty and cytotoxicity challenge was restricted. Natural killer cell-derived exosome (NK-Exo) combine crucial targeted cellular therapies of NK cell therapies with unique non-toxic Exo as a self-origin shuttle against cancer immunotherapy. This review study covers cytokines, adoptive (autologous and allogenic) NK immunotherapy, stimulatory and regulatory functions, and cell-free derivatives from NK cells. The future path of NK-Exo cytotoxicity and anti-tumor activity with considering non-caspase-independent/dependent apoptosis and Fas/FasL pathway in cancer immunotherapy. Finally, the significance and implication of NK-Exo therapeutics through combination therapy and the development of emerging approaches for the purification and delivery NK-Exo to severe immune and tumor cells and tissues were discussed in detail.

## Background

Natural killer cells, as a component of innate immunity, comprise a large number of granular lymphocytes. These cells have been known as the most efficient immune cells responsible for immune surveillance because, unlike other lymphocytes such as T-cells, they are not limited by the expression of major histocompatibility complex (MHC) or human leukocyte antigen (HLA) in the target cells and can clear the transformed or infected cells without priming [1].

NK cells establish lytic machinery, which can migrate toward the inflammation sites through various chemoattractants and destroy target cells independently from a priori activation [2]. Therefore, they play a predominant role in restraining cancer hallmarks, including tumor growth and metastasis diffusion and defense against various pathogens [3]. So that, cancer risk can be increased due to the low activity of NK cells in peripheral blood [4].

Great endeavors are being made to understand the sufficient viability, better growth, and proper activation of NK cells to be utilized in cancer immunotherapy (CI) Exos with acidic desire [5, 6].

Among immune system cells, NK cells, derived from CD34^+^ hematopoietic progenitor cells [7] in the bone marrow (BM), have attracted more attention. These cells are generally present in the blood, liver, spleen, BM, and lymph nodes. However, inflammatory factors in the tumor microenvironment (TME) can stimulate NK cells to migrate to most tissues. These cells do not need pre-immunization for their cytotoxic effect, and their subsets have been long-lived. They can generate memory-like responses under certain stimulus conditions. For this reason, NK cells are considered one of the bridges between innate and adaptive immunity [8]. Furthermore, NK cells have been remarked as anti-tumor vaccines in CI due to their ability to rapidly detect and lyse tumor cells with limited effect on other body cells [9]. On the one hand, NK cells derived from peripheral blood can be recognized by CD56 expression within the lymphocyte gate CD3 negative. They can be further divided into cytotoxic CD56-dim-CD16-bright, which contains 90% of NK cells presented under physiological conditions, and CD56-bright-CD16 + included 10% of NK cells generated interferon-gamma (IFN-γ) [10].

In recent years, the different subtypes of extracellular vehicles (EV), including cell membrane-derived microvesicles (MV) (150–1000 nm), apoptotic bodies (1000–5000 nm), and especially Exos (50–150 nm) [11], which are released through immune cells, has been proposed as a cell-free substitute in CI following features [12, 13]. First, the potential of passive tissue diffuses due to the leaky vasculature of the tumor; second, Exos have a desire for acidic environments; so they can remain at acidic TME, which promotes Exos fusion to malignant cells. Furthermore, they can cross biological barriers because of their nano size. Finally, Exos are stable at − 80 °C for about a year, leading to efficient storage and use as an "off-the-shelf" treatment [5, 14]. On the other hand, NK cells can release soluble agents through the encapsulation process in the nanometer-sized EV [15]. Exos act as critical mediators for cell-to-cell communications in various physiological conditions and cancers and primarily function in metastasis, cancer progression, and immune responses [16]. Besides, immune cells can release Exos with tolerogenic and stimulatory features into the extracellular environment and support the potential of immune cells-derived Exos in cancer therapies [17]. To this end, there is a growing interest in applying Exos as biomarkers due to their detailed exploration of biological fluids. Like, as newly reported for the programmed death-ligand 1^+^ Exos in the patients who suffered from melanoma [18]. Efforts to raise a desire for precision medicine are focused on the development of therapies based on the promotion response of innate and adaptive immune systems [19]. Exos can modulate selected cellular activities, such as vascular homeostasis and antigen presentation [20], and demonstrate low immunogenicity and high transport efficiency establishing the application of Exos in CI [21].

Immune cells, including dendritic cells (DC), T- cells, and NK cells, -derived Exos may play a crucial role in cancer immunomodulation [22]. Exos with antigenic peptides induce anti-tumor CD8^+^ T cell responses. To this end, DC-derived Exos can inhibit tumor cells and NK-Exos-mediated surveillance of the primary tumor and, therefore, can inhibit the abnormal proliferation of tumor cells [23]. Subsequently, to explore the use of Exos for the development of NK therapy in personalized medicine, NK-Exos is an innovative art in CI. This review gives an overview of the barriers and advantages of NK-based CI, including the use of cytokine-based therapy and improve the functional performance of adoptive NK cell immunotherapy, along with the manipulation of NK-Exos as a shuttle for regulation and stimulation of the immune system. The study discusses the development and feature of NK-Exos with target markers for CI.

## NK cells in CI: barriers and advantages

Unlike other lymphocytes, NK cells do not express antigen-specific receptors. Instead, they express genome-encoded stimulatory or inhibitory receptors [24, 25]. NK cells’ effector functions are regulated by signals from these activating and inhibitory receptors to avoid potentially dangerous effects on the host [26]. Major activating receptors on NK cells include natural cytotoxicity receptors (NCR) (NKp30, NKp44, and NKp46), C-type lectin receptors (CLR) (CD94/NKG2D, NKG2C, NKG2E/H, and NKG2F), NK-associated CLR (NKp65, NKp80), low-affinity IgG receptor FcγRIII (CD16), SLAM (a group of type I transmembrane receptors) family receptors (2B4, SLAM6 and SLAM7), killer cell immunoglobulin-like receptors (KIR) (2DS and 3DS subtypes), IgSF member DNAX accessory molecule-1 (DNAM-1) and CD137 (4-1BB) [27].

The inhibitory receptors recognized self-MHC-I molecules, including 2DL and 3DL subtypes and CLR such CD94/NKG2A/B. Furthermore, some immune checkpoints (ICP) such as programmed death-1 (PD-1) [28], cytotoxic T-lymphocyte-associated protein 4 (CTLA-4) [29], T cell immunoglobulin, and mucin domain containing-3 (TIM-3) and T cell immunoreceptor with Ig and ITIM domains (TIGIT) receptor, conduct inhibitory signals. Generally, HLA-I molecules (HLA-I) expressed in healthy cells pair with KIR or CD94/NKG2A/B on NK cells to inhibit the killing of NK cells [30].

Tumor cells undergo surface antigenic changes to escape immune responses. For instance, HLA-I is lost or down-regulated in neuroblastoma. Some others activate NK cell receptors’ ligands, which can simultaneously inhibit T and NK cells [31]. The lack of HLA-I expression on tumor cells (“missing-self” recognition hypothesis) eliminates the inhibitory signal delivered through KIR or CLR receptors [32].

Immune stress increases damage-associated proteins in tumor cells. These proteins, such as MICA/MICB, ULBPs, B7-H6, BAT3, CD155, and CD112, can couple with NK cells activating receptors and initiate the cytotoxicity function of NK cells. NK cells simultaneously received activating and inhibitory signals. In the next step, the integration of these signals determined the result of NK cell activation [33].

Activated NK cells can apply their cytotoxic effect on the malignant cell directly and indirectly. Several mechanisms are involved in the direct method, including the release of lysis lysosomes with perforin (PFN) and granzyme (Gzm), which induce apoptosis due to the death receptors’ characters. Among these death receptors, Fas (CD95/APO-1/TNFRSF6) -associated death domain (FADD) is essential for apoptosis induction which is triggered by Fas-FasL (ligand) attachment. The interaction recruits FADD and binds procaspase-8, thus forming the death-inducing signaling complex (DISC) and caspase-8 active effector caspase-3 [34, 35]. Other receptors include tumor necrosis factor (TNF)-related apoptosis-inducing ligand (TRAIL) and antibody (Ab)-dependent cell-mediated cytotoxicity (ADCC) induction which is usually mediated by CD16 (FcγRIIIa). At the indirect path, NK cells modify immune system cytotoxic activity by secreting cytokines, especially IFN-γ, chemokines such as CCL4 and CCL5, and growth factors like granulocyte–macrophage colony-stimulating factor (GM-CSF) and adenosine [36].

NK cells also exhibit non-specific antigen-trained immunity (memory and memory-like functions) in the innate immune system, which is classified and studied in three categories hapten specific, virus-specific, and cytokine-induced [37]. Cytokine-induced memory-like (CIML) NK cells are attractive cell types for adoptive cellular immunotherapy strategies, especially CI [38]. Human NK cells exhibit memory-like attributes after brief pre-stimulation with IL-12, IL-18, and IL-15 cytokines [39]. In these cells, CD25 expression increased, KIRs and TGF-β expression decreased, and INF-Ɣ production increased after restimulation. Also, epidemic changes like CpG demethylation, metabolic changes, especially in glucose transporter and transferrin receptor toward glycolysis, in vivo enhanced proliferation capacity, cytotoxic granule components change, and in vivo long-term persistence have been reported. These features may cause inhibition release, enhance anti-tumor responses, and long-term persistence and recall functions of CIML NK cells [37, 40, 41].

**Fig. 1:**
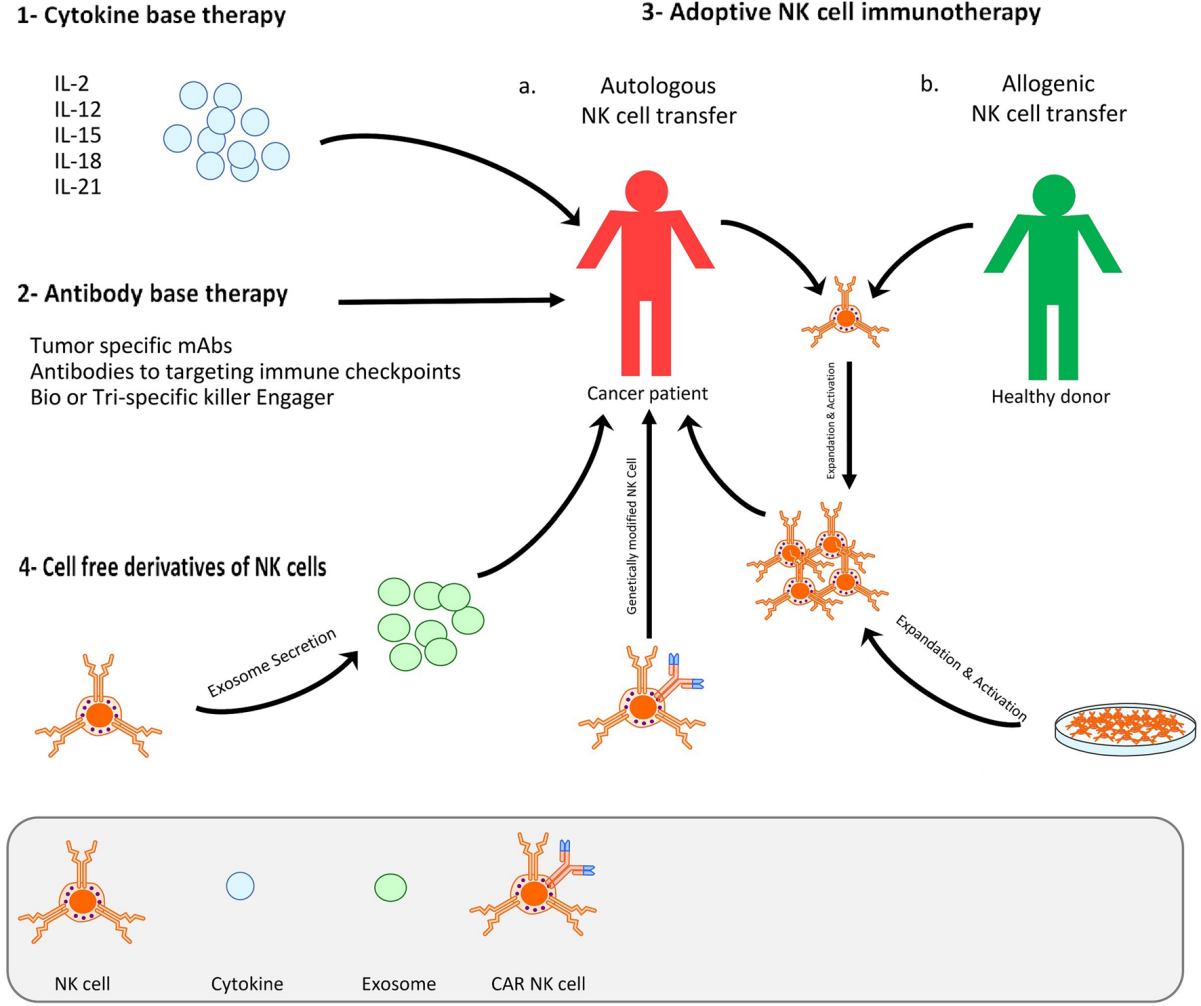
NK cell immunotherapy strategies. Different strategies for NK cell immunotherapy. First, treatments with cytokines to promote NK cell proliferation and activation; Second, treatment with antibodies or inhibitory receptors for NK cell and ADCC activation or facilitate NK cell cytotoxic activity; third, adoptive NK cell immunotherapy (autologous or allogenic NK cell transfer); fourth, treatments with cell-free derivations (Exos) of NK cells for overcome direct cells using limitations

The presence of NK cells at the first line of immune responses for quick response to transformed and stressed cells [42], along with some features such as the rapid release of inflammatory cytokines, action on killer cells without the need for pre-immunization, acquisition of immunological memory under certain conditions, and the existence of numerous sources, such as autologous, allogeneic, and peripheral blood mononuclear cells, stem cells, cell lines, and genetically modified NK cells to obtain them has led to an increasing number of NK cell-based CI studies [43, 44]. Several approaches used for NK cell-based cancer therapy include cytokines, Ab, the adoptive transfer of unmodified NK cells, adoptive transfer of NK cell lines, the adoptive transfer of genetically modified NK cells, and the use of cell-free derivatives of NK cells [45, 46]. Figure 1 represents efficient strategies of NK cell immunotherapy. In the first strategy, patients are treated with stimulatory cytokines, which boost NK cell proliferation and activation to better anti-tumor responses. Second, Abs either activate NK cells and ADCC or facilitates NK cell cytotoxic activity by inhibiting NK cell inhibitory receptors. In the third strategy with an adoptive NK cell immunotherapy, NK cells (autologous, allogenic, cell line or CAR NK cell) are ex vivo activated and expanded, then transfused into the patient to improve immune responses. Finally, in the last approach, Exos used as a cell-free CI to overcome direct cells using limitations.

## Strategies of NK cell immunotherapy

### Cytokine-based therapy

Cytokines can be used to boost NK cell responses against malignant cells. Their action mechanism is through direct or indirect stimulation of NK cells. Directly, cytokines improve the autologous NK cell numbers, differentiation, and activation in vivo. The second pathway is a preliminary step in preparing NK cells for use in adoptive transfer. In this method, NK cells are incubated with one or a mixture of cytokines after proliferation and activation in vitro [45]. Several clinical trials have evaluated the efficacy of effective cytokines on NK cell responses, including IL-2, 12, 15, 18, and 21 [47]. Initial clinical trials for the treatment of malignant cells recruited used IL-2-activated NK cells [48]. Cytokine-activated apoptosis in NK cells and their systemic administration toxicity are the main limitations of cytokines used with NK cells in CI. It seems that cytokine combinations, IL-2 or IL-15 with IL-12 or IL-8, increase NK cell apoptosis related to their activation level due to the synergistic effect [49]. A therapeutic strategy to overcome these limitations is using cytokines with synergistic anti-tumor effects and incompletely overlapping toxicities in combination with pulse dosing in CI. Similarly, influential growth factors (such as Flt3-L, SCF, and IL-7) have been suggested in the early stages of NK cell differentiation along with NK activating cytokines. These growth factors can also increase immature and mature NK cell numbers [50]. The role and function of efficacious cytokines in NK cell therapies, with their advantages and drawbacks, were summarized in Table 1.

**Table 1:** The role and function of efficacious cytokines at immune paths of NK cell therapy

Cytokine	Advantages	Difficulties	Other forms	Refs.
IL-2	First FDA-approved cytokine promotes NK cells in vivo homeostasis, cytotoxicity, and proliferation Repeated injections are well tolerated at low doses and produce LAK cells	Activation of T_reg_ Life-threatening toxicity at high doses, including vascular leakage and organ injury	Super-2 (mutant form): increased affinity to IL-2Rβ, independent of IL-2Rα (CD25), reversed NK cell’s anergic state, promoted NK cell proliferation, and the expansion of cytotoxic T cells but not T_reg_	[5, 8, 75]
IL-15	Substantial role in homeostasis and cytotoxicity of NK cells. Activate both immature and mature NK cells and memory CD8^+^ T cells without activating T_reg_ In combination with IL-12 and IL-18 generate CIML NK cells	Fever, thrombocytopenia, and hypotension	dsNKG2D-IL-15: protein fusion and enhanced NK cell-targeting ability through increasing tumor growth-suppressing capacity IL-15 (heterodimeric): chimeric protein fusion of IL-15 with IL-15Rα promote NK cells and represses tumors	[37, 45, 51, 75, 157]
IL-12	Stimulate the NK cell production of cytokines (particularly IFN-γ) In combination with IL-15 and IL-18 generate CIML NK cells Robust NK cell anti-tumor immunity by enhanced cytotoxicity effect	–	NHS-IL12: Modified cytokine (IL-12^+^ tumor necrosis-targeting human IgG1), prolonged half-life, reduced side effects, and activating splenic and tumor-infiltrating NK	[37, 45, 51]
IL-18	Increase NK cell activity and differentiation and induce memory NK cell formation NK cells survive through C-apoptosis inhibitor 2 and TNF receptor-associated factor 1 pathway In combination with IL-12 and IL-15 generate CIML NK cells	–	–	[37, 45, 54, 158]
IL-21	Enhance IFN-γ production and cytotoxic functions Limited viability support limit expansion and start apoptosis of NK cells	–	–	[137, 146, 159]

### Adoptive NK cell immunotherapy

There are three types of NK cell-based Ab treatments: tumor-specific Ab, Ab to target ICP for release inhibition of NK cells, and bi- or tri-specific killer engagers to potentiate NK cell engagers (NKCE). To use tumor-specific Ab*,* tumor-specific monoclonal antibodies (mAbs) recognize the tumor-related antigens on the surface of tumor cells. These mAbs attack malignant cells by different mechanisms, including delivering toxic molecules to target cells and killing targets through promoting NK cells (ADCC) over interactions with CD16 (FcγIIIA) activating receptors [51, 52]. In the second approach*,* NK cells prevent overactivation and express ICP receptors such as PD-1, CTLA-4, TIM-3, LAG-3, CD96, IL-1 receptor 8, KIR, and CD94/NKG2A. The ligands of these receptors were expressed by malignant cells to escape the immune system. Therefore, using mAbs that inhibit these receptors, for instant, anti-PD-1, and anti-PD-L1, release the brake on cell growth and enhance the anti-tumor activity of NK cells [53]. Finally, NKCE consists of a single-chain variable fragment (scFv) designed to form an antigen-immunological synapse between activated NK cells and tumor cells to boost NK cell tumor targeting and cytotoxicity [4, 54]. Adoptive transfer of NK cells is an approach to replacing, repairing, and improving the immune system functions through infusing autologous, allogenic cell lines and chimeric antigen receptor (CAR) consists of an extracellular antigen recognition domain fused to an intracellular signaling domain that redirects the specificity and function of immune cells [55] NK cells are a suitable and promising cell for adaptive immunotherapy due to their unique anti-tumor properties [56].

### Adoptively transferred NK cells

In NK cell adoptively transferred strategies, diverse and innovative approaches are used for isolating, expanding, manipulating, or producing ex vivo activated autologous or allogeneic NK cells, which are administrated to patients to act against the tumor [57]. These cells are isolated or differentiated from multiple sources such as peripheral blood of patients or healthy donor, stem cells (induced pluripotent stem cells, human embryonic stem cells, or hematopoietic stem cells), and established human cell lines and then expanded with different methods to sufficient and large scale[58, 59]. One of the challenges for successful NK cell therapy is the ability to obtain large numbers of cells from a limited number of NK cells from different sources [60]. Research is ongoing to improve methods to overcome these hurdles. For instance, fresh NK cells are traditionally used in treatments, but nowadays, cryopreservation of mature NK cells may be possible. In the expansion phase, different practical protocols combine serum-free media, supplements, recombinant cytokines, stimulatory antibodies, small molecules, pharmaceutical products, and irradiated feeder cells. For clinical procedures, animal serum products don’t use, in addition, tissue culture flasks have given way to bioreactor systems or culture bags in dedicated GMP facilities. Feeder cells also play an important role in the expansion of NK cells by providing cell-to-cell communication [61]. Although research continues to find new methods to reduce dependence on feeder cells and increase the efficiency of NK cell expansion, for example, the use of two heterodimeric multi-cytokine fusions for NK cell proliferation and activation [62].

### Autologous adoptive transfer of unmodified NK Cells

Early trials using NK cells as an adoptive cellular therapy for cancer treatment occurred in the 1980s. These trials were based on the delivery of lymphokine-activated killer (LAK) cells [63]. However, their clinical outcomes were poor, with severe side effects because of IL-2 high dose usage [64]. Clinical trials showed autologous NK treatment was safe and universal but faced limited efficiency due to the transfer limited on tumor suppression [65]. Likewise, contributing to NK cell killing could be inhibited because of self-HLA-I expressed by tumor cells and recognized with NK cell-KIR or direct NK cell cytotoxicity by IL-2-induced regulatory T cells (T_reg_) [66]. Thus, strategies such as using anti-KIR Ab to block NK cell inhibitory receptors interacting with their HLA-I ligand on target cells have been developed to overcome these limitations [52].

### Allogenic adoptive transfer of unmodified NK cells

Owing to the limitations of autologous NK cells, using allogeneic NK cells from related healthy donors instead of autologous cells in adoptive transfer therapies was developed. These allogeneic cells are educated in a non-immunosuppressive environment to become fully functional before transfer. In addition, these cells could be used as allogeneic off-the-shelf products for instant clinical use [67]. The most crucial hazard of allogeneic cell therapies is graft-versus-host disease (GVHD) development. Howbeit, as opposed to T cells, allogenic NK cells, especially haploidentical NK cells, do not induce GVHD due to a lack of surface T cell receptors (TCRs) on NK cells which improve transfer efficiency and decrease related toxicity [68, 69]. Allogeneic NK cell therapies, especially in the metastatic phase, have been clinically evaluated, and there is still no standard protocol or product [68, 69].

### Adoptive transfer of genetically modified NK cells

CAR NK Compared to other CAR cells (CAR T) exhibited improved tumor-specific targeting and cytotoxicity in both in vitro and in vivo settings and featured with targeted immunotherapy. At first, since the allogeneic NK cells are not aroused GVHD, unlike CAR T cells, they do not require HLA matching. Second, CAR NK does not have clonal expansion; therefore, they do not induce cytokine storms. On the other hand, the NK cells mainly released IFN-γ and GM-CSF; thus, their cytokine production profile was considered safe. Third, because NK cells are obtained from various sources such as peripheral blood, umbilical cord blood, human embryonic stem cells, induced pluripotent stem cells, and even NK cell lines, CAR NK production is cheaper and time-saving than CAR T cells [70–72]. Eventually, CAR cells have a long-term persistence, which increases the risk of autoimmunity or malignancy because NK cells are short-lived. CAR NK cells are expected to disappear rapidly after the intermediation of their anti-cancer effects, and they do not need a "suicide system" to clear the cells [73]. Despite such benefits of CAR-expressing NK cell production, it also faces problems such as difficult isolation and expansion of large numbers of NK cells, especially from peripheral blood, the sensitivity of these cells’ cryopreservation, and low transfection efficiency. Therefore, attention has been attracted to NK cell lines because NK cell lines are composed of fully activated NK cells, are easy to transfect, and can expand well under appropriate production conditions [74, 75].

Only the NK-92 cells have been approved by the FDA for clinical applications among the recognized NK cell lines. NK-92 cells’ safety and feasibility have been confirmed by different clinical trials [76]. NK-92 is a pure allogeneic activated NK cell source, IL-2 dependent, represented cytotoxic against various malignancies, and has no KIR [77]. These cells are more simply proliferated than donor-derived primary NK cells (under GMP conditions), making them cost-effective [78]. In addition, they can be manipulated with various vectors to increase their targeting, homing, and cytotoxic activity. Despite these advantages, the efficacy of the NK-92 cell remains limited for several reasons, like limited life span, low effectiveness, and the need to be irradiated before injection to entirely revoke their proliferation because of tumorous tissue origin, which severely affects their persistence in the body. Although repeated injections appear helpful, they stimulate the immune system against the HLA expressed in NK-92 cells, which increases the removal of these cells [79].

### Cell-free derivatives from NK cells

Despite many advances in the use of NK cells in CI, the efficacy of direct cells has been moderate at best. Effectiveness limitation reasons include limited transferred NK cell persistence in the body, restricted migration and penetration ability into tumor tissues compared to other immune system cells, the evolution of NK cell escape mechanisms in tumor cells, and finally, NK cells inhibition by TME component amongst T_reg_, myeloid-derived suppressor cells (MDSC) that cause NK cells function inhibition and transforming growth factor-β (TGF-β), adenosine, prostaglandin E2 (PGE2) and indoleamine 2,3-dioxygenase (IDO) which have been associated to NK cell dysfunction [80].

## Therapeutic potential of exosome

**Fig. 2:**
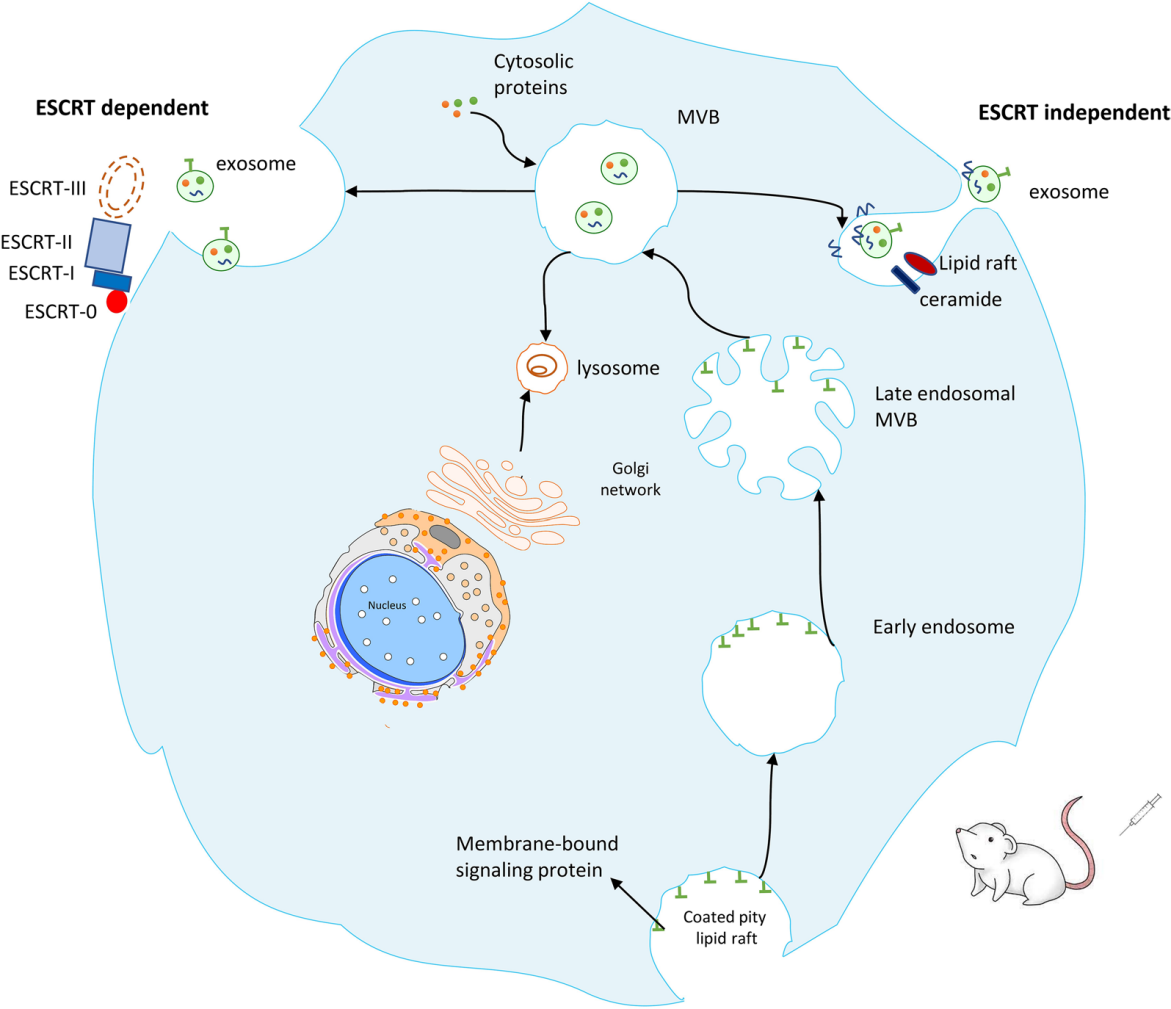
Exosome biogenesis process. The whole procedure of exosome biogenesis from source to delivery. Start with the internalization of proteins and lipid complexes; then continued with the inward budding of endosomes which pack and transport cargoes inside multivesicular bodies (MVB). finally, Exos are released from the cells which can be used for treatment after isolating and characterizing. NK, natural killer; IL, interleukin; mAb, monoclonal antibody; CAR NK, chimeric antigen receptor-natural killer

Exos play a dual role in dealing with pathogens and the pathogenesis of infections. Furthermore, Exos derived from infected cells can deliver Pathogen-associated molecular patterns (PAMPs), especially at intracellular pathogens, to surrounding cells and stimulate antimicrobial immune responses [81]. Exos are created by endosomes' inward budding, which packed and transport cargoes inside multivesicular bodies (MVB) [82] (Fig. 2). Hallmarks of Exos biogenesis are first an endocytic event at the plasma membrane. After the maturation of early endosomes to the late endosomes, intraluminal vesicles (ILV) are formed by inward budding of the endosomal membrane, which gives rise to MVBs [83]. MVB usually turns into lysosomes (Degradative MVB) where proteins aimed for destruction are enriched. However, some MVBs (Exocytotic MVBs) take an alternative route, moving to the plasma membrane, and by fusing with it, they release their ILV cargo as Exos in the extracellular space [84].

At least two distinct pathways mediate the process of exocytotic MVB biogenesis and involves the sorting of various molecules into ILV [85]. The first pathway leading to MVB formation requires the endosomal sorting complex required for transport (ESCRT). The first model suggests that Exos are formed within the endocytic pathway in a two-step process and are released from the plasma membrane via MVBs in the extracellular environment, such as inner vesicles [86]. In fact, in this way, Exos originate by the inward budding of the endosomal compartment membranes, forming intraluminal bodies, using the machinery of the endosomal ESCRT [87]. This hetero-oligomeric protein complex (ESCRT-0, -I, -II, and -III) is critical in MVBs formation, sorting, and secretion. ESCRT-I, -II, and -III recognize monoubiquitinated cargoes and possibly GPI-anchored, then promote their inclusion in MVBs. Once completed, the ESCRT complex dissociates from the MVB membrane aided by the adenosine triphosphatase vacuolar protein sorting 4 (Vps4) and is recycled for subsequent cargo [88].

The second pathway of secretion is the ESCRT-independent mechanism. In this method, proteins such as the transferrin receptor are present in ILV but are not ubiquitinated. These proteins, which lack the sorting signal for ubiquitination, are partitioned into the ILV based on their intrinsic physical properties and preference to segregate into raft-like microdomains [86, 89]. Studies demonstrated that several Rab family proteins (GTPases), including sub-family Rab27, 35, and 11, act as crucial Exos transport and secretion regulators in these ways. Together with calcium, they were shown to participate in the docking and fusion of MVB with the plasma membrane [90, 91]. In this regard, Exos display has allowed us to create recombinant vesicles carrying cytokines or tumor antigens that may or may not have been previously present on Exos [92]. This technology is used to induce epitope-specific Ab response against tumor biomarkers. This Ab method called ExoMAb gives us new opportunities to design new therapies and generate novel diagnostic tools [93].

### Immune system stimulatory associated with Exos

**Fig. 3:**
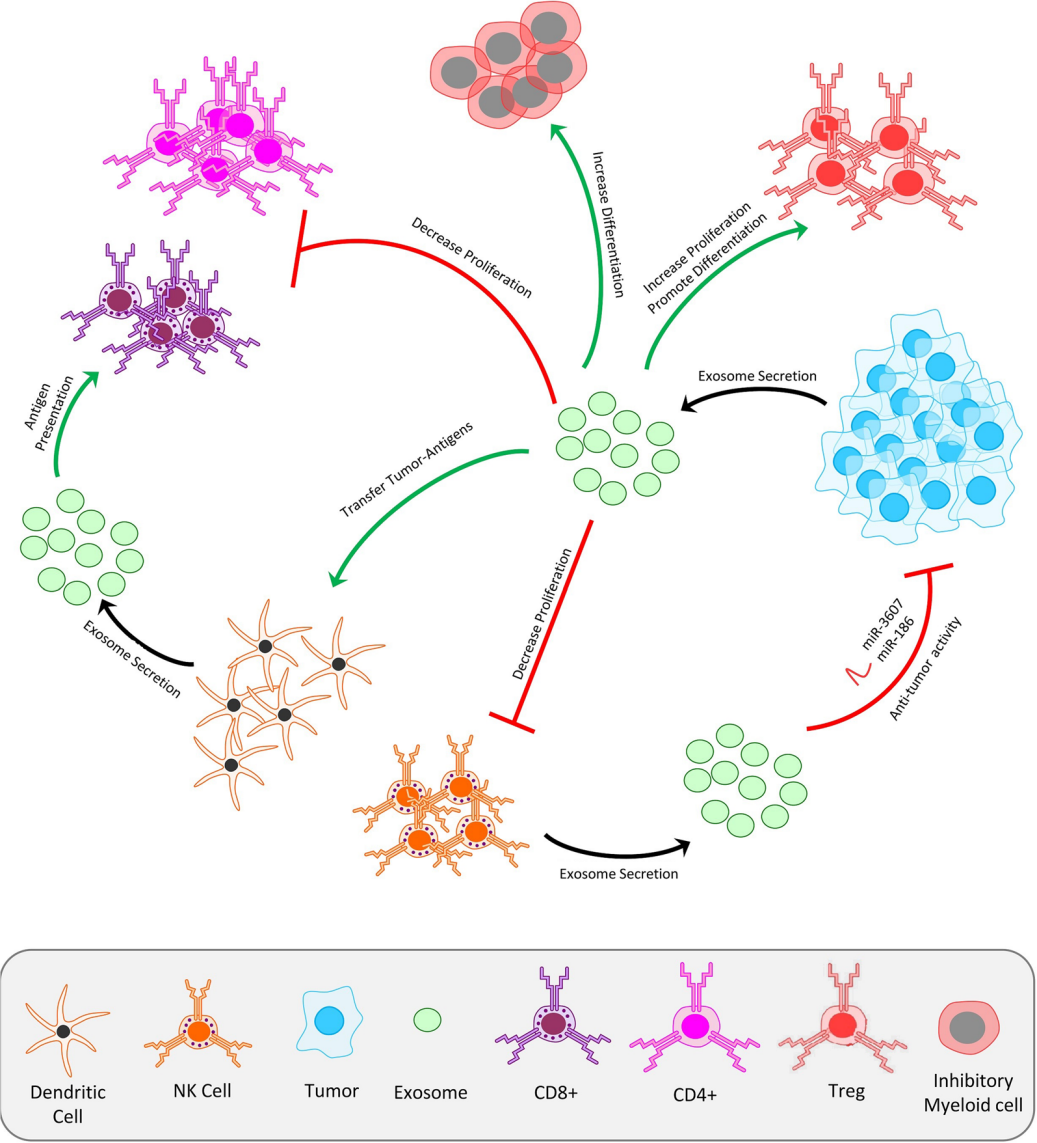
Immunomodulatory effects of exosomes on the immune system. In antigen-specific immune responses, Exos spread and presented originated cells antigens which increased the number of DC cells presenting these special antigens. In the following this DC’s derived-Exos (Dex) induce anti-tumor immune responses by interact with CD4^+^ and CD8^+^ T cell. tumor-derived Exos may have immunosuppressive effects such as NK cell cytotoxic activity inhibition, decrease T lymphocytes proliferation, increased inhibitory myeloid cells’ differentiation and promotion of T_reg_ expansion and differentiation

DC-derived-Exos (Dex) have functional MHC-I-peptide[94] complexes that could induce CD8^+^ T cell-dependent anti-tumor immune responses in mice. This finding and other similar results form the basis of the Exos active roles in the intercellular communications hypothesis, at least in the immune system. [95, 96]. During antigen-specific immune responses, Exos can increase the number of DC presenting them (Fig. 3) or directly interact with memory T cells by spreading origin cell (pathogen-infected cells, tumor cells, and inflammatory cytokines expressing cells) antigens or presenting them with MHC-peptide complexes. The outcome of this spreading depends both on the state of the DC which captures Exos (especially for Exos from non-stressed or noninfected immature DC or tumors) and on molecules carried by Exos (e.g., pro-inflammatory signals from mature/infected/stressed cells or immunosuppressive signals from some non-stressed tumors) [97].

T-cell activation analyses by Exos have shown that MHC-peptide complexes in Exos directly bind to their cognate T-cell receptor and activate primed CD4^+^ and CD8^+^ T cells (Fig. 3). On the other hand, naive T lymphocytes need DCs that have captured Exos and present the Exosomal MHC-peptide complexes to specific T cells. This difference could be because of the activated conformation of lymphocyte function-associated antigen (LFA-1) integrins at the surface of primed (but not naive) T lymphocytes, which allows effective binding of intercellular adhesion molecule-1 (ICAM1)-bearing Exos to these T lymphocytes as it does to LFA-1-expressing DC. It also reflects that naive T cells need cytokines secreted by DC and the TCR-dependent signal for activation. These immune effects can be used in various therapies, such as anti-cancer [98].

In addition to Exos immune-stimulant roles, Exos has also demonstrated the ability to encourage tolerance in the immune system, as shown in Fig. 3. If Exos secreted by immature DC or DC subjected to immunosuppressive treatments or modified to express immunosuppressive cytokines injected alone, it promote tolerogenic immune responses, which could be helpful as treatments for autoimmune diseases [99].

For instants, before transplant surgery, Exos introduced to a patient from a donor beget longer transplant acceptance time in the patient [100]. On the other hand, tumor-derived Exos may have immunosuppressive molecules on their surfaces, such as (membrane-associated) TGF-β1 [101] or FasL, which may cause DC differentiation inhibition from myeloid precursors, inhibition of NK cell cytotoxic activity, inhibition of T lymphocytes and promotion of T_reg_ expansion that leads to immune system evasion or induces an antiproliferative effect [102]. Furthermore, some experiments showed Dex-mediated reduction of NK T cells, which are unrelated to Treg actions [103].

So the tumors derived Exos exhibit contradictory immune effects; sometimes they can decrease the proliferation of CD4 and CD8 T lymphocytes/NK cells or promote the differentiation of immunosuppressive cells such as T_reg_ or myeloid cells and, at other times, Exos from tumor cells promotes tumor growth and metastasis by increasing the differentiation of inhibitory myeloid cells and decreasing NK cell activity. Other tissues and cells secrete Exos with immunosuppressive molecules. For instance, placenta-derived vesicles have FasL-mediated T-cell inhibiting properties or Prostasomes in semen. Placental explants also secrete Exos that inhibit NK lymphocytes. Moreover, Exos present in milk and colostrum exhibits immunosuppressive effects on T cells. Exos present in the bronchoalveolar fluid of the lung and intestinal, depending on the host state, can show tolerizing features or conversely increase pro-inflammatory cytokine secretion by airway epithelial cells [104].

The application of Exos in CI was developed based on the antigen-presenting cell (APC) to DC, which can activate specific CD4 + and CD8 + T cells and NK cells [81]. Tumor-derived Exos (isolated from tumor cells or DC pulsed with tumor antigens) induces the anti-tumor response by carrying tumor antigens, delivering them to T cells, and priming them, which causes tumor cell death. This ability proposed that tumor-derived Exos can be useful in developing a cell-free cancer vaccine [89]. DC-Exos in cell-free anti-cancer vaccine clinical trial phase I demonstrated the ability to activate NK cell proliferation and activation. Such observations promise that a cell-free anti-cancer vaccine will be available shortly [105].

### Isolating and characterizing NK cell-derived exosomes methods

Different protocols can be used for Exos purification. The basic and most common protocol is sucrose density gradient ultracentrifugation which contains multiple centrifugation and ultracentrifugation steps[106]. This method is very time-consuming and requires a large biological sample volume. The final product has high purity with no protein contamination. Sometimes initial steps are replaced by a single filtration step, which reduces time and cost, but a large amount of initial sample is the problem [107, 108].

The second purification method employs beads with specific antibodies for exosomal surface molecules which trap Exos (Immunoaffinity capture-based techniques). The efficiency of this method is high for separating specific subgroups of Exoes. However, the required reagents for this protocol are costly and can only be used in cell-free samples [108].

The third procedure involves ultrafiltration cartridges and pumps which are useful for purifying Exos from large, and medium volumes, which makes it suitable for clinical applications of purified exosomes [109]. Although ultrafiltration produces highly-purified Exos, removing adherent protein from Exos- membrane is complex, and material shape and electrical charge effet on ultrafiltration procedure [107].

The fourth method is Microfluidic isolation devices which reduce sample size and save time by carrying out multiple steps simultaneously [107]. Therefore The development of new methods is always considered; for example, recently, Kang et al. developed a streamlined microfluidic chip that combined microfluidic devices and chemical release strategies for harvesting NK-Exos from viable NK cells [110].

Exos identification and characterization involved morphological analysis with an electron microscope or Nanoparticle tracking analysis (NTA), Exos purity evaluation, separation by SDS-PAGE, exosomal proteins staining, physical properties analysis on a continuous sucrose gradient, protein quantity, and composition by immunoblotting or Enzyme-linked immunosorbent Aasay (ELISA), and Flow cytometry analysis[109, 111].

## Performance of NK-derived exosomes in cancer and cancer Immunotherapy

### Cytotoxicity and anti-tumor activity

NK-Exos specified cytotoxicity and anti-tumor activities over a range of cancers. For instance, the therapeutic efficacy of NK(92MI)-Exos revealed considerable cytotoxicity activity against glioblastoma [112] and melanoma [113]. In contrast, NK-Exos co-cultured with K562-mbIL21 cells exhibited anti-cancer activity against breast carcinoma and acute lymphoblastic leukemia (ALL) [114].

**Fig. 4:**
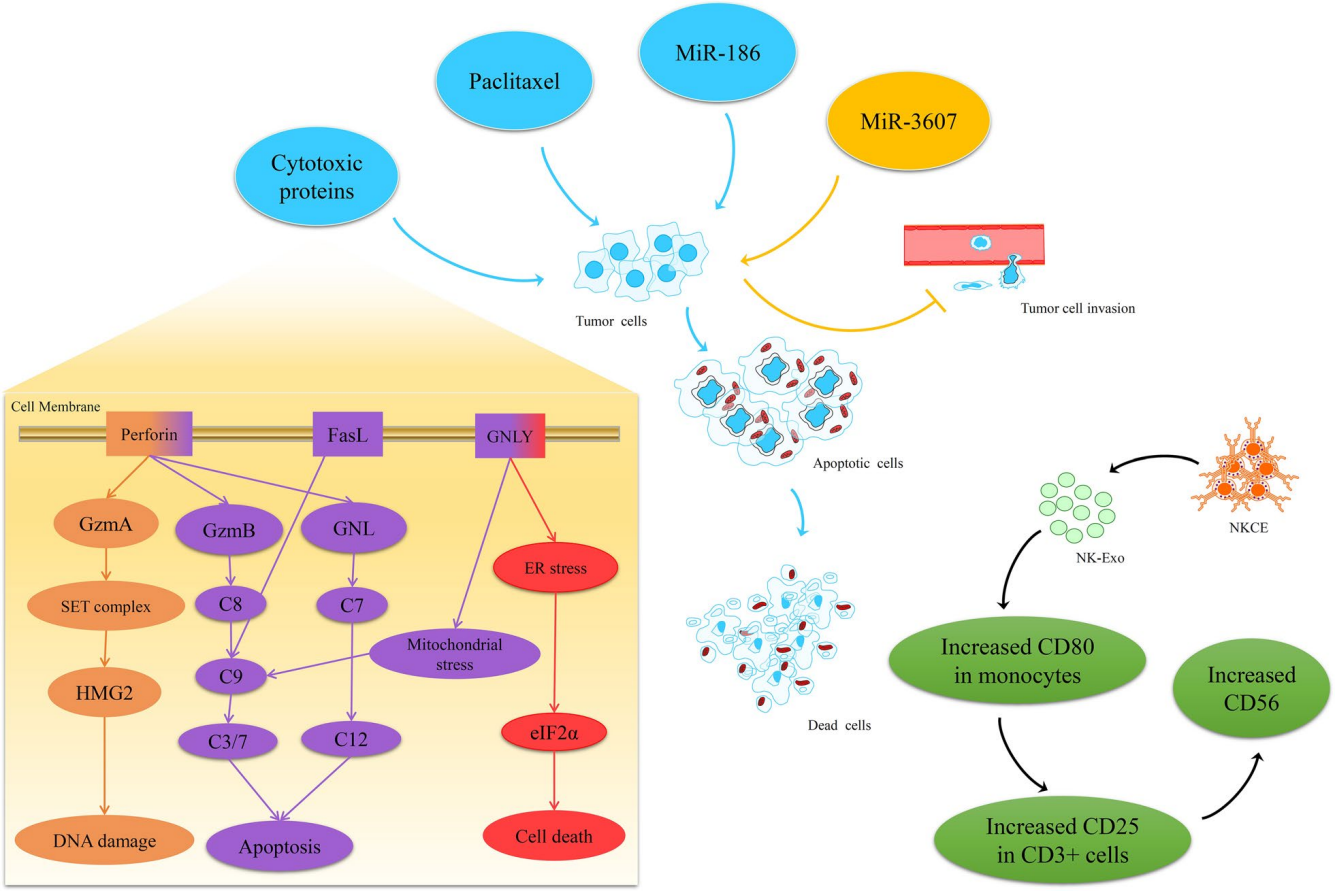
The roles of NK-Exos in immunostimulatory (modulation), immunosuppression, and cytotoxic activities. NK-Exos exert their effects through different mechanisms depending on bioactive molecules including therapeutic drugs, miRNAs (MiRs), and cytotoxic proteins which carried. Paclitaxel (PTX) loaded-Exos, kill tumor cells; MiR-3607 loaded-Exos, prevent invasion of cancer cells; MiR-186 loaded-Exos, suppressing cell proliferation and inducing; cytotoxic proteins loaded-Exos, kill cancer cells by caspase-dependent and -independent apoptotic pathways

NK-Exos exerts its effects through different mechanisms due to bioactive molecules, including therapeutic drugs, miRNAs (MiRs), and cytotoxic proteins [115]. MiRs are a family of naturally occurring small noncoding RNAs act as oncogenes or tumor suppressors [116–119]. These bioactive molecules' function, amount, and type can vary depending on the cell pretreatment, physiologic status, or source. No single molecule is involved in the cytotoxic efficacies of NK-Exos [115]. Figure 4 shows Paclitaxel (PTX) loaded-Exos can kill breast tumor cells [120, 121]. Furthermore, NK cells release Exos consisting of MiRs. MiR-3607-3p in NK-Exos prevents the further invasion of pancreatic cancer cells via targeting IL-26. MiR-186 in NK-Exos could down-regulate aurora A kinase (AURKA) and v-myc avian myelocytomatosis viral oncogene neuroblastoma-derived homolog (MYCN) expression, thereby suppressing cell proliferation and inducing apoptosis in neuroblastoma cells [122].

Moreover, Fig. 4 represents the cytotoxic proteins’ performance in killing cancer cells by caspase-dependent and -independent apoptotic pathways. Granulysin (GLN) damages the cell membranes and activates stress-mediated apoptosis. Granzyme B (GzmB) directly induces procaspase and interrupts mitochondria, releasing cytochrome c and inducing the apoptotic cascade. Granzyme A (GzmA) induces the cleavage of the SET protein complex, resulting in single-stranded DNA damage [123]. Finally, NK-Exos stimulates immune cells, which in turn includes five modes of action, including stimulating the expression of co-stimulatory molecules on monocytes, activating T cells via acting on monocytes, directly activating T cells through up-regulation of CD25, stimulating NK cells and enhancing the percentage of CD56dim NK cells in the presence of T cell and monocyte stimuli [124].

### Caspase-independent/dependent apoptosis

PFN, a pore-forming protein, allows Gzm in NK-Exos to enter host cells and generate a pore in the endosome, releasing Gzm. The entrance of NK-Exos into the host cell triggers caspase-independent and -dependent apoptosis [125]. In caspase-independent cell death, GzmA actives mitochondrial stress, causing the reactive oxygen species release and DNA damage. This protein also can target nuclear proteins for degradation, leaving deoxyribonucleic acid vulnerable to caspases and nucleases [126]. In caspase-dependent cell death, GzmB directly induces a signaling cascade that interrupts mitochondrion and releases cytochrome C, triggering caspase-7, -3, and -9 [127, 128]. GLN directly harms the cellular membrane and triggers ER stress-derived apoptosis. It was reported that activation of the caspases, the release of cytochrome C, accelerated degradation of SET nuclear proto-oncogene, and high-mobility group protein 2 (HMG-2) in NK-Exos-treated tumor cells. The changed expression of ER-related markers, including phosphorylated eukaryotic initiation factor (eIF2α) and R-like ER kinase, revealed that ER stress is responsible for the processes as mentioned above [129]. Therefore, ER, mitochondrial stress, and caspase signaling are critical in NK-Exos-activated cytotoxicity.

### FasL/Fas pathway

FasL, a type II transmembrane protein of the death factors, activates poly (ADP-ribose) polymerase and caspase-3 and -8, triggering the exogenous apoptotic pathway. This protein is packed into NK-Exos [130]. The membrane-bound and soluble forms of FasL transmembrane protein have been reported in most NK-Exos [131, 132]. They act through different mechanisms such as the endocytic pathway, whereby NK-Exos consisting of soluble FasL proteins are taken up by the cell and active FasL-derived cell death and receptor-ligand interactions, which displayed dose and time-dependent toxic efficacies on melanoma [75, 113]. However, the function of FasL in NK-Exos-derived toxicity remains controversial, with most of the information achieved from anti-Fas Ab blockade studies. For instance, NK(92)-Exos indicated cytotoxic effects on breast cancer (MDA-MB-231/F) and melanoma (B16F10) cells that were abolished via anti-Fas Ab [114, 133]. In contrast, although NK-Exos consists of FasL, they might not be associated with the toxicity of NK-Exos. It has been suggested that the FasL/Fas pathway is affected by Fas expression on the target cell and NK-Exos [67].

### Immunomodulatory and immunosuppression

NK cells exert immunomodulatory effects by releasing Exos, consisting of molecules targeting the immune system [134]. NK-Exos can stimulate immune cells and induce T cells either indirectly or directly by increasing the expression of co-stimulatory molecules on monocytes, thereby activating T cell proliferation [135]. NK-Exos were also reported to induce NK cells, predominantly those of the CD56bright phenotype [136]. Moreover, NK cells subjected to NK-Exos were found to change the natural cytotoxicity receptors (NCR) expression. They had higher toxicity against neuroblastoma cells [137].

Immunosuppression is a mode of action by which cancer cells escape the immune responses through the continuing secretion of Exos and soluble factors or through compelling the expression of ICP molecules [135]. NK-Exos can regulate the immune responses or even reverse cancer immune suppression, making them candidates for CI [135]. TGF-β impedes the recognition and clearance of cancer cells through immune cells [138] and acts along with lipopolysaccharide or IL-10, for mimicking an immune-prohibitive environment. However, TGF-β increases the anti-tumor capacity of NK-Exos [139]. Furthermore, these Exos stimulated NK cells, T cells, and monocytes even under immune-preventative conditions. Several reports have suggested that NK-Exos possess substances acting on the TGF-β pathway, thereby alleviating immune suppression [140–142]. NK-Exos can decrease the expression of the primary ICP molecule, which is expressed by various immune cells and presents a critical function in immune escape by cancers [143] on CD28/CD3-induced T cells. Besides, NK-Exos consists of various molecules associated with the immune response, such as MHC-II, MHC-I, IL-10, TNF-α, and IFN-γ. However, their efficacies have not been well-known [22, 144–146].

### Recent advances in the application of NK-Exos for cancer immunotherapy

The distinctive properties of Exos derived from specific immune cells, including NK cells, offer a solution to several difficulties arising from the complication of TME. Their abundance and nano-size are desired for cancer therapy by efficient trafficking to solid tumors and penetration into TME [131]. NK-Exos penetrates problematic barriers, such as the blood-tumor, and blood–brain barrier (BBB), which are relatively impenetrable to NK cells [147]. Besides the penetration of NK cells into cancers, including solid tumors, their Exos strongly affected by the cytokine profile of TME, which occasionally damages NK cell activity [137, 148]. The cell-to-cell transferability of Exos provides an opportunity to communicate with surrounding cells or stimulate other TME activity, leading to cytotoxic effects [149]. Anti-tumor performance of NK-Exos against various cancers indicates the significant potential of this strategy for CI. Table 2 summarizes the targeted Abs (markers) with the result of NK-Exos treatment approach for CI. To date, some studies have focused on the applications of NK-Exos in clinical practices[150].

**Table 2:** Application of NK-Exos used in CI with targeted Abs (markers) and features

Antibody (markers)	Target	Result	Refs.
CD56, Rab5B, CD63, NKG2D, NKp30, NKp46, NKp44, FasL and PFN	Jurkat (T cell leukemia), K562 (erythroleukemia), DAUDI (Burkitt lymphoma), SKBR3 (Metastatic breast adenocarcinoma), and 501mel (Metastatic melanoma)	NK-Exos reveals significant controlling immune responses and defense against tumors and infected cells	[154]
CD81, CD63, TSG101, NKp44, CD69, PD-1, Gzm A and B, NKp46, IFN-γ, LFA-1, CD16, NKG2D, DNAM1, PFN, NKp30	NALM-18 (Childhood B-ALL) and K562 (Erythroleukemia)	Effective combination immunotherapy of NK-Exos with an efficient cytotoxic effect on tumor cells	[111]
Actin, Myosin, ERM proteins, Cytosolic GAPDH, HSP60, HSP70, MHC-I, MHC-II, CD9, CD63, CD81, CD82, Tsg101, and CD56	Human healthy donor NK cells and PBMCs	NK-Exos plays a promising role in supporting NK-mediated immunosurveillance and increasing NK-mediated immune responses against tumor cells	[135]
CD63, GM130, Alix, Cytochrome-c, PFN, FasL, and β-actin	NK92-MI, human glioblastoma (D54), breast carcinoma (MDA-MB-231), anaplastic thyroid cancer (CAL-62), hepatic carcinoma (HepG2) cell lines, and xenograft mouse tumor model	Exos mimetics (NK-EM) have tumoricidal effects and targetability against tumor cells	[160]
CD63, ALIX, GM-13, β-actin, FasL, and PFN	B16F10 (Melanoma) cells, Kidney Phoenix ™ -Ampho cells, and NK-92MI	NK-Exos exert high cytotoxic effects on melanoma cells	[113]
CD56, TSG101, ALIX, NKp30, NKp44, NKp46, KIR2DL1 and NKG2D	SK-N-SH and CHLA-255 (Neuroblastoma)	NK-Exos cells demonstrate higher toxicity and lethality against tumor cells than naive NK-Exos	[137]
CD63, Alix, GM130, GzmB, Calnexin, PFN, and FasL	In vivo: (BALB/c) In vitro: U87/MG(/F) glioblastoma, and NK92-MI	Evidence of NK-Exos antitumor effects in both in vivo and in vitro models on glioblastoma cells	[112]
ALIX, TSG101, CD63, CD47, CXCR4, and Cytochrome C	CHLA-255 (Neuroblastoma), MDA-MB-231 (Breast cancer), and embryonic kidney cell lines 293 T	NN/NK-Exos cocktail has efficient cancer targeting and MiR tumor cells’ delivery	[145]
TSG101, Alix and CD63	Human NK-92 cells and MCF-7 (Breast cancer)	PTX-NK-Exos increased the apoptosis of tumor cells by increasing the expression of Caspase-3 and Bax	[21]
CD56 and CD63	NK-92MI cell line and lung cancer clinical samples	NK-Exos on-chip biogenesis show high cytotoxic effects	[110]
HIF-1α, FasL, PFN, and GzmB (functional markers)	MCF-7 (Human breast cancer), A2780 (Human ovarian adenocarcinoma), NK92-hIL-15, and NK92 (NK cells)	Hypoxia enhanced NK-Exos decrease tumor cells proliferation	[14]
BCL-2, CD63, and CD56	NK92M (NK cells), HEK293T (human embryonic kidney), K562 (Bone marrow), MEC-1 ( B-chronic lymphocytic leukemia), MCF-7, SKBR3, MCF-10A, T-47D, and MDA-MB-231 (breast cancer)	NK-Exos loaded anti-Bcl-2 siRNA induces apoptosis in breast cancer cells	[156]
GD2, CD56, CD16, CD3, and CXCR4	CHLA-136, LAN-5, and CHLA-255 (Neuroblastoma)	NK-Exosomal MiR-186 (suppressor) indicates cytotoxicity effect on neuroblastoma cells	[139]
CD63 and TSG10	Mia PaCa-2, and PANC-1(Primary pancreatic cancer)	NK cell Exosomal MiR (3607-3p) inhibits pancreatic cancer by targeting the IL-26	[147]

Zhu et al. investigated the anti-tumor effect of NK-Exos on four tumor types, including glioblastoma, breast carcinoma, anaplastic thyroid cancer, and hepatic carcinoma in vivo and in vitro. NK-Exo-treated glioblastoma xenograft mice had high anti-tumor activity by reducing cell viability markers (p-AKT and p-ERK) and inducing pro-apoptotic proteins. They also found that NK-Exos significantly inhibited the growth and proliferation of glioblastoma (U87/MG) cancer cells [112]. To this end, Shojae-Hassani et al. characterized and tracked the effect of NK-Exos cells on tumor cells after incubation to overcome the resistance of malignant cells to immune responses. NK-Exos exposed to neuroblastoma cells could activate Exos of the naive NKs population to exert a more severe cytotoxic effect on target cells [137].

Moreover, Zhu et al. demonstrated that NK-Exos expressed two vital proteins of NK cells such as FasL and PFN could affect the signaling pathway of tumor cell proliferation by overexpression of TNF-α against invasive melanoma in vivo. NK (92MI)-Exos specified two functional proteins of NK cells, FasL and PFN; furthermore, it secretes TNF-α, which influences the signaling pathway of cell propagation [113]. Accordingly, Federici et al. indicated that NK-Exos induced the expression of CD25 on T cells, co-stimulatory molecules on monocytes, and human leukocyte antigen DR (HLA-DR) exhibited stimulatory function in peripheral blood mononuclear cells (PBMC) in the blood of melanoma patients. NK-Exos enhanced the CD56^+^ NK cell fraction, proposing that effects modulated by NK-Exos may be specified in support of CI. Their findings also recorded lower levels of TSG101^+^CD56^+^ Exos and NK cells in PBMC, highlighting the capacity of an immune enzymatic test to sense alteration of the NK cell-mediated immune system [135].

Lee et al. presented experimental evidence that canine NK-Exos can express specific markers such as Granzyme B, Perforin 1, TSG101, HSP70, Alix, CD81, and CD63. The authors explored the anti-tumor impact of NK-Exos on murine mammary tumors. They observed the tumor size reduced and the apoptotic markers such as Bcl-xL and Bax, as well as tumorigenesis-associated markers such as PCNA, p53, MDR, TNF-α, IL-1β, MMP-3, VEGF, and Bmi-1 were declined. Thus, they suggested that canine NK-Exos present a potent therapeutic agent against mammary carcinoma tumors [137, 151]. Dosil et al. found that NK-Exos microRNAs, such as miR-155-5p, miR-92a-3p, and miR-10b-5p, can target specific molecules responsible for Th1 response. NK-Exos down-regulated GATA-3 transcript in CD4^+^ T cells. They led to the polarization of Th1 and the accumulation of IL-2 and IFN-γ. NK-Exos microRNAs partially recapitulate NK-EV impact in vivo. This observation provides novel insight into the immunomodulatory role of NK-Exos towards improving its application as an immunotherapeutic tool in cancers [122, 137]. Nie et al. observed that NK-Exos not only can induce tumor apoptosis directly but also promote the action of cytotoxic T lymphocytes by multiplex pathways, including reprogramming tumor-related macrophages and up-regulating MHC-I in tumor cells. As a result, the authors suggested that NK-Exos can remarkably enhance adoptive T-cell therapy against solid tumors by immune-modulatory functions [137, 152].

To evaluate NK-Exos in leukemia, a group of hematologic cancers was studied on BM, leading to abnormal blood cells. In an endeavor, Boyiadzis et al., during two studies, suggested that NK-Exos-based cytotoxicity against leukemic targets presents a new therapeutic approach for leukemia patients. In this research work, NK-Exos are used to carry PD-1, KIR, TGF-β, GzmB, PFN, natural cytotoxicity receptors, and NK cell receptor NKG2D and determine the dose of used NK-Exos. NK-Exos mediated anti-leukemia activity against primary leukemia blast, acute myeloid leukemia (AML), and the K562 cell line with an escalating dose of NK-Exos (10–70 µg) [149]. Notably, a higher level of cytotoxicity was recorded with a higher dose of NK-Exos, suggesting that Exos-modulated lysis is controlled in a concentration-dependent manner [153].

The potential of NK-Exos in homeostasis and immune surveillance of CI is encouraging and may become the most efficient cancer vaccines and drug/antigen carriers. Thus, understanding how NK-Exos can be used in immunotherapy as a novel player for the suppression of cancer progression. In a study, Lugini et al. demonstrated Exos purified from healthy donor plasma could express NK cell markers, such as PFN and CD56^+^, and apply cytotoxic activities against various activated immune and cancer cells. They suggested a crucial role of NK-Exos in homeostasis and immune surveillance [154].

Moreover, Di Pace et al. evaluated the Exos from cytokines-cultured NK cells to reveal the mechanisms of Exos-based immunotherapy in human cancer. IL-15 and IL-2 induced comparable levels of Exos with similar cargo composition. Analysis of molecules exposed at the surface or contained within Exos permitted the recognition of molecules playing a vital role in the NK cell function, such as PD-1, DNAX accessory molecule-1 (DNAM1), LFA-1, and IFN-γ. Based on the observations, DNAM1 is responsible for Exos-mediated toxicity, which delays host cell apoptosis. NK-Exos can diffuse into tissues and exert its catalytic efficacy at the tumor site. This offers a clue to integrate cancer treatments with NK-Exos [111]. Wu et al. also monitored the levels of cytotoxic proteins from NK-Exos, including FasL (2.5 ng/mL), granulysin (GNLY, 56 ng/mL), GzmB, (23.4 ng/mL), GzmA (185 ng/mL), and PFN (550 ng/mL). The association between cytotoxicity and cytotoxic protein levels revealed that GNLY, GzmB, GzmA, and PFN are all positively associated with toxicity, proposing that no single toxic protein is predominantly responsible for killing and that all these proteins might contribute to toxicity. In cells treated with NK-Exos, the GzmA substrates, HMG2 and SET, decreased, demonstrating that this protein may activate a caspase-independent death pathway. Besides, the main marker of the caspase-dependent death pathway, i.e., cytochrome C, was released from mitochondria. The authors indicated that several killing mechanisms are induced by NK-Exos, including caspase-dependent/-independent cell death pathways, which can modulate toxicity against tumor cells [129].

Li et al. also presented evidence regarding MiR-containing NK-Exos alleviated signs of chronic mild stress in mice. In vivo*,* the assessment revealed that these NK-Exos declined the levels of pro-inflammatory cytokines IL-6, TNF-α, and IL-1β released by astrocytes. MiR-207 directly targeted TLR4 interactor through leucine-rich repeats, suppressing NF-κB signaling in astrocytes and decreasing anti-depressant activities. As a result, the authors suggested that MiR-207-containing NK-Exos can alleviate signs of depression in mice by targeting leucine-rich repeats to hinder NF-κB signaling in astrocytes [155].

Wang et al. reported a new strategy called cocktail therapy with the combination of biomimetic core–shell nanoparticles (CSNPs) with NK-Exos. The CSNPs/ NK-Exos cocktail via FasL/Fas and endocytosis are highly efficient carriers for MiR delivery to neuroblastoma cells [145]. Accordingly, Han et al. showed that the rate of migration and apoptosis on MCF-7 (breast) tumor cells treated with NK-Exos encapsulated with PTX (PTX-NK-Exos) decreased and increased significantly, respectively [21]. Recently, Tae Kang et al. used the NK-graphene oxide (GO) microfluidic chip method to isolate NK-Exos from the blood of patients with non-small-cell lung carcinoma (NSCLC). This new isolation method provides a new perspective of NK-Exos with high cytotoxic function against circulating tumor cells [110]. Additionally, in another study, Jiang et al. evaluated the function of NK (92)-Exos with hypoxia NK (92-hIL-15)-Exos and NK (92)-Exos significantly, which increased the available protein content of NK cells such as GzmB, PFN, and FasL. They suggested that hypoxic treatment of NK-Exos cells could be a helpful treatment option for cancer treatment [14].

In a study, Kaban et al. evaluated a new strategy based on NK-Exos to target the Bcl-2 gene dose in breast cancer cell lines. Their results reported that NK-Exos loaded with anti-Bcl-2 siRNAs significantly reduced Bcl-2 expression and induced apoptosis. [156]. Neviani et al. reported that NK-Exos carrying tumor suppressor MiR-186 had high cytotoxicity against MYCN-amplified neuroblastoma cell lines. Their data showed that MiR-186 expression was significantly reduced in patients with neuroblastoma. They also found a negative relationship between the expression of MiR-186 and the expression of TGF- β2, TGF-β1, AURKA, and MYCN genes, which play an essential role in the progression of neuroblastoma cells. Targeted delivery of MiR-186 to neuroblastoma cells could significantly inhibit the progression of neuroblastoma cells. It could be used as a complementary therapy in combination with NK cell-based immunotherapy [139]. Accordingly, Sun et al. examined the ability of cancer cells for migration, angiogenesis, and proliferation in pancreatic tumor cells cultured simultaneously with NK cells. The biotin-RNA pull-down assay and reporter assay data showed that NK-Exos-loaded miR-3607-3p could target the IL-26 expressed in the pancreatic TME, inhibiting growth and the development of pancreatic tumor cells in vitro and in vivo. They suggested these Exos could be a suitable approach to treating various cancers [147].

## Conclusion

Manipulation of NK-Exos could be deemed a promising approach for CI. In addition to the ability for targeting of nerve cells (glioblastomas, neuroblastomas, and leukemia), immune cells (T/B-chronic lymphocytic), and severe tissue (brains and BMs), NK-Exo can effectively be employed as a targeted strategy against various cancerous conditions. By optimization of NK-Exo with emerging extraction and purification technologies (such as microfluidics) and improvements in innate and adaptive immune systems, personalized medicine can be achieved. Thus, rapid evolution of precise NK-Exo monitoring assays with the ability to follow and analyze single cells in CI, opens up new possibilities in the field of targeting, diagnosis, and detection. Finally, NK-Exo combined with synergic effect of other (drug) agents acts as a combination therapy, which could pave the way to overcome the challenges and dilemmas of CI.

## Data Availability

Not applicable.

## Abbreviations

FDA: Food and Drug Administration
T_reg_: Regulatory T cells
LAK: Lymphokine-activated killer
IFN-γ: Interferon-gamma
TNF: Tumor necrosis factor
MYCN: V-myc avian myelocytomatosis viral oncogene neuroblastoma-derived homolog (MYCN)
C12: Caspase 12
C9: Caspase 9
C8: Caspase8
C7: Caspase 7
C3/7: Caspase3/7
GzmB: Granzyme B
GzmA: Granzyme A
PFN: Perforin
GNLY: Granulysin
AURKA: Aurora kinase A
TME: Tumor microenvironment
MV: Microvesicles
DNAM1: DNAX accessory molecule-1
ALL: Acute lymphoblastic leukemia
PBMCs: Peripheral blood mononuclear cells
NSCLC: Non-small-cell lung carcinoma
